# Online physician recommendation with dynamic capacity and semantic similarity: A primal-dual optimization framework

**DOI:** 10.1371/journal.pone.0357449

**Published:** 2026-09-15

**Authors:** Yan Qiu, Yi Chen, Yinghong Xie, Min Li, Junhui Yan, Hu Liu

**Affiliations:** 1 School of Economics and Management, Jiangsu University of Science and Technology, Zhenjiang, Jiangsu, China; 2 School of Business, Anhui University, Hefei, Anhui, China; 3 Department of Pathology, First Affiliated Hospital of Soochow University, Suzhou, China; 4 School of Economics and Management, Wuhan University, Wuhan, Hubei, China; 5 School of Management, Hefei University of Technology, Hefei, Anhui, China; 6 School of Economics and Management, Southeast University, Nanjing, Jiangsu, China; International Institute of Information Technology Bangalore, INDIA

## Abstract

Existing physician recommendation methods mostly start from the patient’s perspective on online healthcare platforms, neglecting the importance of physician service capacity and semantic similarity. Considering the uncertainty of arrivals and capacity constraints, this study aims to propose a dynamic capacity and semantic similarity-based framework to recommend physicians. We design an online rolling matching algorithm based on delayed decision-making. We employ MPNet, a pre-trained language model based on masked and permuted language modeling, to extract contextual semantic representations from unstructured consultation records and construct physician-patient matching quality. The proposed method is validated using a real-world dataset with 88,009 records from one of the largest online healthcare platforms in China. The results show that our method significantly outperforms baseline methods. It achieves competitive ratio of approximately 0.988 on both CIM and HS datasets, with the largest improvement reaching 0.094 over the baseline methods. The improvements over all baselines are statistically significant. The sensitivity and robustness analysis further demonstrate that the integration of dynamic capacity constraints and MPNet-based semantic similarity enhances the effectiveness and stability of online physician recommendation. This study provides a reliable reference for users to find online physicians and offers practical implications for the design of online healthcare platforms. The implementation of our method is available at https://github.com/qiuyan-just/dynamic_matching to facilitate reproducibility.

## Introduction

Physicians are a scarce medical resource, due to the high-quality requirements, large educational investment, and long training time, which makes it challenging to meet the growing demand [1]. Online healthcare platforms have the potential to mitigate insufficient total amount and uneven distribution of high-quality medical resources [2]. It expands the boundary of physician-patient interaction [3,4] and enables physicians to realize greater value by utilizing fragmented time [5]. This necessitates a rational allocation of physician resources based on the performance of recommendation systems. Providing accurate recommendations can guide patients to seek medical treatment in an orderly fashion [6]. How to match physicians with patients on online healthcare platforms has gradually emerged as a research focus.

However, the uncertainty in real-world settings brings tremendous pressure for recommendations. Most traditional distributional models assume that the potential probability distribution of patient arrivals can be fully estimated using real data or that we possess some prior knowledge. Such models are hard to effectively allocate resources to hedge future arrivals [7]. Due to unforeseen factors in practice, it is unrealistic to accurately estimate the arrival distribution. Stochastic optimization and robust optimization are two important ways to solve optimization problems in uncertain environments [8]. The medical field lacks sufficient data to accurately estimate the arrival distribution of patients. In addition, robust optimization uses uncertainty sets rather than distributions to characterize uncertainty. Although it can be more robust than stochastic optimization, it is heavily dependent on the structure of the uncertainty set. Online optimization has been shown to be robust to uncertainty in dynamic environments [9]. Therefore, this study employs an online matching approach to address the uncertainty.

Physician resources are limited by their service capacity [10]. Recommendations that exceed the workload of physicians are of low value. Notably, one study found that online matching between physicians and patients is subject to dynamic capacity constraints [11] and revealed that physicians are in different capacity states. Therefore, achieving dynamic physician recommendations requires considering both the uncertainty and dynamic capacity constraints of physicians. On the issue of physician recommendations based on healthcare data, many studies have carried out a lot of effective work from different perspectives, mainly focusing on patient preference [12,13], trust [14], and privacy protection [15]. Data sparsity makes it challenging to mine useful information. The pre-trained language model has become an important tool for obtaining fine-grained features. The introduction of Transformer makes the subsequent pre-trained model structure more complex, such as Bidirectional Encoder Representations from Transformers (BERT), Robustly Optimized BERT Pretraining Approach (RoBERTa), and masked and permuted language modeling (MPNet for short) models. These models can generate context-based semantic representations with the help of attention mechanisms, significantly improving the effectiveness of many downstream tasks [16]. Therefore, leveraging the characteristics of question-answering pairs, we train semantic features to better calculate the matching quality [17].

In this study, we focus on an online physician allocation problem with capacity constraints. We construct a linear programming (LP) model under the primal-dual framework, taking into full account dynamic capacity constraints, and the matching quality between physicians and patients. The objective function is to maximize the reward. However, in practice, not all arriving patients can be assigned to the right physician at once. To break through this dilemma, we further expand the physician-patient matching algorithm based on delayed decision-making, which is called online rolling matching. This algorithm allows for the postponement of allocation for patients not arranged on the first day to subsequent days, continuing until each patient is matched with a suitable physician at the earliest opportunity.

We aim to propose a dynamic physician recommendation that considers the uncertainty and service capacity in online healthcare platforms. Our research goals can be summarized as:

- To explore how to establish a general online physician-patient matching framework that incorporates capacity constraints and matching quality for dynamic recommendations.
- To study how to design an online rolling matching algorithm that can provide a patient allocation plan and determine service times.
- To enhance recommendation performance by improving the measurement of physician-patient matching quality

By recommending physicians to patients considering dynamic capacity constraints and semantic similarity, this study bridges gaps in research with the following primary contributions:

- We introduce an online rolling matching method leveraging machine learning and primal-dual optimization for dynamic physician recommendation. The proposed method integrates estimated capacity constraints and semantic compatibility while incorporating a replenishment mechanism in online setting with unknown patient arrivals.
- We combine contextual text embedding with LP to solve optimal matching problems. From a fine-grained perspective, we utilize semantic features to compute the matching quality.
- We validate the proposed method on one of the largest online healthcare platforms in China and the performance of our method significantly outperforms existing baseline models.

## Related work

Considering patient preference and selection behavior, previous studies have built optimization models to achieve physician recommendations [18,19]. Additionally, an optimal or near-optimal matching algorithm between buyers and sellers has been proposed for generating recommendations [20]. This study aims to construct an online matching model from the perspective of physician resources to complete dynamic recommendations. This section will comprehensively review three streams of relevant works: online matching, primal-dual approaches, and semantic similarity.

### Online matching

Online matching refers to an uncertain dynamic environment where one party arrives in real time and the other party is waiting to be matched. Matching is achieved through specific strategies for arriving and waiting parties. It can help find matches that align with user needs and selection preferences. Online platforms such as ride-sharing [21–25] and crowdsourcing [26–28] have greatly benefited from optimal online matching. Existing research has focused on the following topics.

In the traditional matching market, the vertices on one side are fixed and have known a priori information, while the vertices on the other side arrive online to be matched by the agent. However, there are conflicts between offline agents in real-life scenarios, such as online recommended books of the same type. For maximizing individual or group-level fairness among offline agents, online matching with stochastic rewards has been studied, employing a new path-based formula and extending the primal-dual scheme [29]. In response to the gap in existing research on which matching strategy to adopt for a specific market, a matching model based on communication complexity theory considering different market characteristics and bilateral participant preferences was established [30]. The impact of bilateral preferences on the design of online algorithms for a matching platform was studied [31]. Subsequently, they proposed an online classification algorithm to maximize the expected number of matches.

Facing the reusable characteristic of offline resources, a non-adaptive algorithm based on LP was put forward to match drivers and potential passengers [32]. The services re-enter the cloud manufacturing platform after being occupied for a period. Online policies with performance guarantees were proposed [33]. A rolling time domain method was proposed to dynamically handle arriving requests [34]. The deferred acceptance algorithm was extended to a discrete-time stable marriage framework with the consideration of uncertainty constraints by insurance types [35]. On the contrary, authors in [36] studied the online weighted bipartite graph matching problem under capacity constraints, where resources are offline non-replenishable. To address the service capacity constraint, a task-expert matching model was constructed to maximize task resolution throughput [37].

### Primal-dual approach

The primal-dual approach is a powerful algorithmic scheme that has proved to be extremely applicable in a wide range of problems in the area of approximation algorithms. It has been extended to the setting of online algorithms. The basic idea is that LP and its dual model guide the decisions made by the online algorithm. A primal-dual learning algorithm to realize personalized pricing over a finite selling horizon has been presented in [38]. In the recommendation field, most methods use auxiliary information based on primal-dual to improve user models and address cold start [39]. An adaptive fairness-aware online meta-learning algorithm that takes into account the primal and dual parameters of the model has been designed in [40]. The Assemble-to-Order (ATO) problems with the general structure and integrality constraints are well-known to be difficult to solve. The primal-dual analysis and an LP model were employed in [41,42]. Optimal transport needs a significant amount of computation and storage space for large-scale problems. The transshipment problem was formulated as a mixed integer linear program in [43]. Other groups in [44] also developed a Lagrangian relaxation with a primal-dual approach to find upper bounds. A primal-dual iterative training algorithm has been designed to learn a policy that satisfies the constraints of ride-sharing platforms. For the data-driven risk-averse newsvendor problems, regularization with the primal-dual approach were combined in [45] to transform into convex quadratic programming problems with good theoretical properties.

### Semantic similarity

Semantic similarity has been applied to many fields, such as online question-answering systems [46], online retrieval [47], representation learning [48] and healthcare recommendation [49]. The wide application of pre-trained language models significantly improves the performance of tasks related to textual semantic similarity [50]. Transformer was employed to measure the semantic similarity between sentences and relationship types [51]. Topic modeling and semantic measures were combined to address redundancy in text summarization [52]. Word representations from the vector space model and Latent Dirichlet Allocation (LDA) were integrated to calculate semantic similarity. The semantic similarity of the BERT model was adopted to enhance the topic recognition ability of LDA [53]. Semantic similarity based on the XLM-RoBERTa Transformer structure was adopted for semi-automated fact-checking [54]. In [55], candidate skills were extracted from resumes and matched with job descriptions. Deep learning was then used to calculate the semantic similarity between the extracted entities and resume text snippets.

Although online matching, primal-dual, and semantic similarity have been widely used in multiple fields, research on online matching based on primal-dual optimization and machine learning for physician recommendation is still lacking. Choice capacity is the key design feature of an online matching platform. Different choice capacities affect the number of choices and match on the platform. In [56], randomized experiments were used to demonstrate that providing users with greater choice capacity has different effects on choice ability and matching outcomes on an online dating platform. This, in turn, verifies that our study’s consideration of physician service capacity is valuable. It is promising to adopt the advanced pre-trained language model to extract semantic information. Therefore, we primarily address dynamic physician recommendations through online matching, taking into account dynamic capacity and complex semantic similarity.

## Methodology

In this section, the overall framework of recommendation is presented first. Then we describe the problem statement for physician-patient matching.

### Theoretical framework

**Fig 1:**
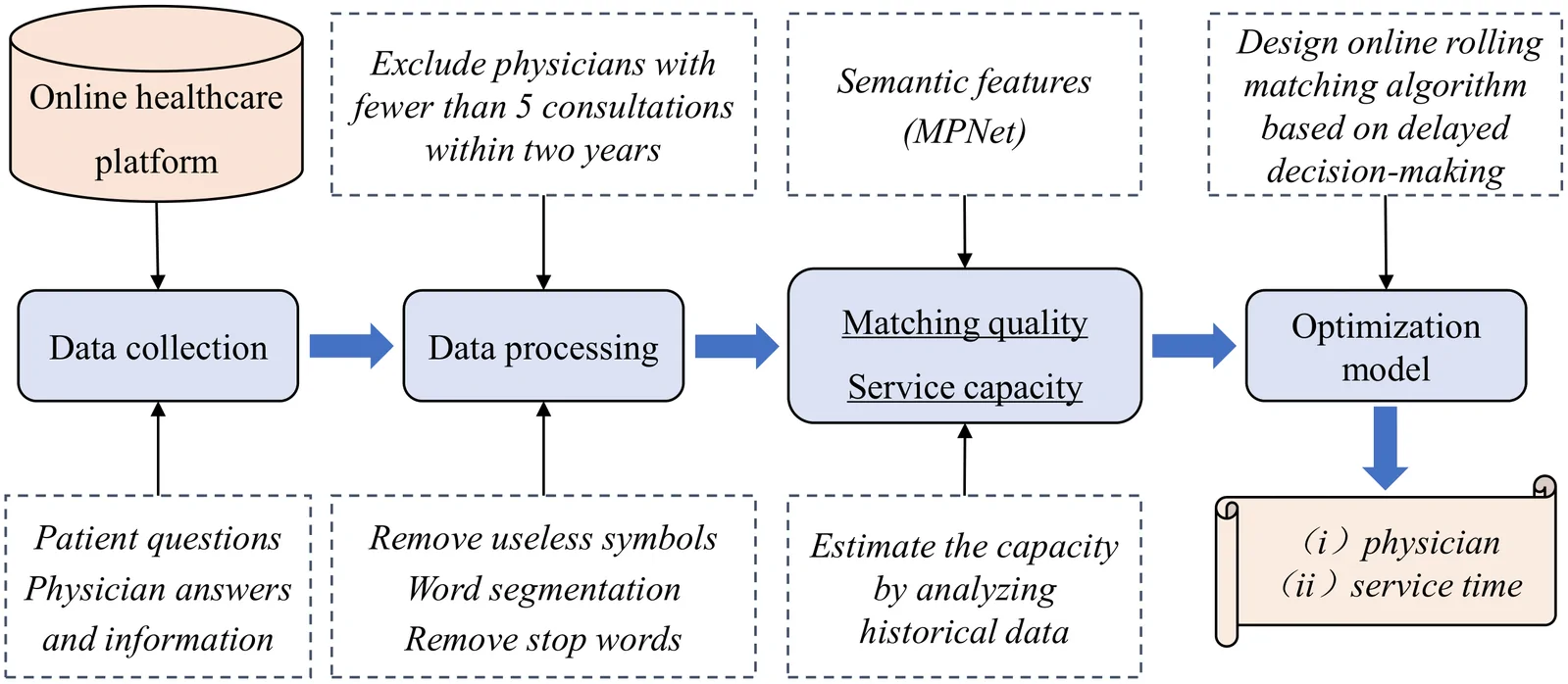
The framework of dynamic physician recommendation.

With the rapid development of online healthcare platforms, the way patients seek medical services has changed substantially. Fig 1 illustrates the dynamic physician recommendation framework. First, patient questions, physician answers, and physician profile information are collected and processed. Then, the MPNet model is employed to extract semantic features, and physician-patient matching quality is calculated using cosine similarity. At the same time, the service capacity of each physician is estimated from historical consultation data. Next, an LP model for online physician allocation is constructed based on patient arrival times and physician service capacities. Furthermore, the corresponding primal and dual models are formulated. Based on delayed decision-making, an online rolling matching algorithm is then designed. Finally, the proposed method is implemented using the matching scores and estimated capacities to recommend suitable physicians and service times.

### Problem statement

Here, we study an online physician-patient matching problem with dynamic capacity constraints. There is no prior information about patient arrivals. The platform will process the request when the patient arrives, and provide matching decisions without grasping the future arrival. Let [m]={1,2,...,m} and [n]={1,2,...,n} to represent the set of m physicians and the set of n patients, respectively. We assume the full information about physicians is already known. T denotes the total scheduling time. For each tϵT, $c_{i,t}$ is the capacity of physician i on time t. For each arriving patient jϵ[n], we can have personal information and disease descriptions. By using this information, the reward of each patient, which is defined as the matching quality $r_{i,j}$ can be calculated. We should realize $r_{i,j}$ are not known in advance, only when each patient j arrives. Once a patient arrives on the platform, the general practitioners perform an online matching immediately without knowing future patient arrivals. Hence, $x_{i,j,t}$ as binary decision variable represents whether a physician i is allocated to a patient j at time t. The goal is to find an optimal matching that maximizes the pairing quality between the set of physicians and patients in a capacity-constrained environment.

### Optimization model

Based on the above descriptions, we formulate the physician-patient matching problem under the primal-dual framework. The online matching can be formulated as the following primal model (P) and the dual model (D).

For primal problem (P):


$$\begin{array}{c} \text{max}\sum_{t=\text{1}}^{T}\sum_{i=\text{1}}^{m}\sum_{j=\text{1}}^{n}x_{i,j,t}r_{i,j} \end{array}$$ (1)

$$\begin{array}{c} s.t.\sum_{j=\text{1}}^{n}x_{i,j,t}⩽c_{i,t},\forall i\epsilon [m],\forall t\epsilon T \end{array}$$ (2)

$$\begin{array}{c} \sum_{t=\text{1}}^{T}\sum_{i=\text{1}}^{m}x_{i,j,t}⩽\text{1},\forall j\epsilon [n] \end{array}$$ (3)

$$\begin{array}{c} x_{i,j,t}\epsilon \left\{\text{0},\text{1}\right\},\forall i\epsilon m,\forall j\epsilon n,\forall t\epsilon T \end{array}$$ (4)

Constraint (2) limits the number of patients a physician can serve each day. Constraint (3) allows a patient to be allocated to only one physician. We intend to get an LP relaxation when we relax the binary constraints to continuous ones in the primal model. We define the dual variables $\alpha_{i,t}$, $\beta_{j}$ corresponding to the above constraints (2) and (3), respectively.

For dual problem (D):


$$\begin{array}{c} \text{min}\sum_{t=\text{1}}^{T}\sum_{i=\text{1}}^{m}c_{i,t}\alpha_{i,t}+\sum_{j=\text{1}}^{n}\beta_{j} \end{array}$$ (5)

$$\begin{array}{c} s.t.\alpha_{i,t}+\beta_{j}⩾r_{i,j},\forall i\epsilon [m],\forall j\epsilon [n],\forall t\epsilon [T] \end{array}$$ (6)

$$\begin{array}{c} \alpha_{i,t},\beta_{j}⩾\text{0},\forall i\epsilon [m],\forall j\epsilon [n],\forall t\epsilon [T] \end{array}$$ (7)

Subsequently, we construct an online algorithm under the primal-dual framework that ensures performance guarantees to solve the online version of the model (P) and its corresponding dual model (D). We adopt the offline model (P) as a benchmark to evaluate the performance. Here, the construction of dual variables ${\hat{\alpha }}_{i,t}$ and ${\hat{\beta }}_{j}$ can define a feasible dual solution. Because of the definition of $\left(i^{*},t^{*}\right)$, we have $r_{i^{*},j}-\alpha_{i^{*},t^{*}}⩾r_{i,j}-\alpha_{i,t}$. Based on the formulation (6), we have


$$\begin{array}{c} \begin{array}{c} {\hat{\alpha }}_{i,t}+{\hat{\beta }}_{j}=\alpha_{i,t}+r_{i^{*},j}-\alpha_{i^{*},t^{*}}\\ \geq \alpha_{i,t}+r_{i,j}-\alpha_{i,t}\\ =r_{i,j} \end{array} \end{array}$$ (8)

### Online rolling matching

This paper aims to present a dynamic physician recommendation method that considers service capacity and uncertainty under an online matching framework. For this purpose, an online rolling matching algorithm is designed, in which the relevant information of each patient is known only upon arrival. Patients sequentially arrive at the platform on the first day t=1 and an irrevocable allocation decision is made upon each patient’s arrival.

Next, the proposed method is introduced in detail. The dual variables $\alpha_{i,t}$ are initialized to 0. Upon a patient jϵ[n] comes in, we will execute an online matching algorithm using the optimal $\alpha_{i,t}$ until all arriving patients are assigned to a suitable physician. We assume that there exists an optimal $\alpha_{i,t}$ in the dual problem. Based on the complementary slackness, the optimal matching $x_{i^{*},j,t^{*}}$ should satisfy $x_{i^{*},j,t^{*}}\cdot (r_{i^{*},j}-\alpha_{i^{*},t^{*}}-\beta_{j})=\text{0}$. In addition, from constraint (6), we have $\alpha_{i,t}+\beta_{j}\geq r_{i,j}$. Further, we can get $\beta_{j}\geq r_{i,j}-\alpha_{i,t}$. The objective of the dual problem is to minimize (5), so we get $\beta_{j}=\text{max}\left\{r_{i,j}-\alpha_{i,t}\right\}$. Therefore, each non-matched patient will be allocated to one physician according to the online matching algorithm, such that $(i^{*},t^{*})=\underset{x\in [m],t\in [T]}{\text{argmax}}\left\{r_{i,j}-\alpha_{i,t}\right\}$.

The dual variables $\alpha_{i,t}$ and $\beta_{j}$ are initially set to zero for ∀i∈[m],∀j∈n,∀t∈[T]. Upon the arrival of the patient j, we can find a candidate physician $i^{*}$ who satisfies the term, which is the matching quality minus the cost of allocating the expected capacity. This term can be viewed as the acceptance or rejection criterion such that a positive value for the candidate physician $i^{*}$ will assign the patient to this physician; otherwise, the patient should be rejected. If the physician $i^{*}$ is recommended to the patient, it means that the capacity will be reduced, and the dual variable $\alpha_{i,t}$ needs to be updated to continue the subsequent physician allocation. For the update of the dual variable $\alpha_{i,t}$, the following rule is adaptively designed as:


$$\begin{array}{c} \alpha_{i^{*},t^{*}}=\alpha_{i^{*},t^{*}}(\text{1}+\frac{\text{1}}{c_{i^{*},t^{*}}})+\eta (\frac{r_{i^{*},j}}{c_{i^{*},t^{*}}}) \end{array}$$ (9)

The first term on the right-hand side of formula 9 increases $\alpha_{i,t}$ by an amount proportional to the fraction of the capacity used by the accepted patients. The second term depends on η and the ratio of the estimated reward to the total capacity.

It is unethical to refuse treatment to patients even if they may not be assigned a satisfactory physician on the same day. To ensure that all patients can get the best medical services, an online rolling matching algorithm under the primal-dual framework is designed. The basic idea is that if we cannot assign a physician to a patient within a fixed time T, the assignment decision will be postponed to the next day, treated as a new arrival, and so on. That is, the algorithm incorporates a delayed decision-making period, and the remaining patients are continuously allocated to the optimal physicians. The algorithm resets the dual variable $\alpha_{i,t}$ to the initial value for calculation. It enables patients who were rejected the previous time to have a greater chance of getting a better physician recommendation in the delayed scheduling period.

**Algorithm 1:** Online rolling matching algorithm based on primal-dual framework.

**Input:** number of available physicians m, length of patient sequences n, service capacity of physicians in time horizon $c_{i,t}$, matching quality $r_{i,j}$, learning rate η.

**Output:** allocation decision $x_{i,j,t}$.

1: Initialize the dual variable $\alpha_{i,t}=\text{0}$，∀i∈[m], ∀t∈[T], $t=t_{\text{1}},t_{\text{2}},...,T$.

2: **for** each arriving patient j **do**

3:  Observe the patient j that arrives sequentially and calculate the matching quality score with all physicians.

4:  allocate patient j to physician $i^{*}$ on time $t^{*}$ such that$(i^{*},t^{*})=\underset{x\in [m],t\in [T]}{\text{argmax}}\left\{r_{i,j}-\alpha_{i,t}\right\}$

5:  **if** $r_{i,j}-\alpha_{i,t}\geq \text{0}$ then

6:   set $\beta_{j}=r_{i^{*},j}-\alpha_{i^{*},t^{*}}$ and$x_{i^{*},j,t^{*}}=\text{1}$

7:   set $\alpha_{i^{*},t^{*}}=\alpha_{i^{*},t^{*}}(\text{1}+\frac{\text{1}}{c_{i^{*},t^{*}}})+\eta (\frac{r_{i^{*},j}}{c_{i^{*},t^{*}}})$

8:  **else:**

9:   $x_{i,j,t}=\text{0}$, $\beta_{j}=\text{0}$,∀i∈[m],∀t∈[T]

10:  end if

11: end for

12: set $\alpha_{i,T+\text{1}}=\text{0}$, $t_{\text{1}}=t_{\text{1}}+\text{1}$, T=T+1 and continue step 2.

The specific steps are provided in Algorithm 1. Assume that there are m available physicians on the platforms, and $c_{i,t}$ represents the number of patients that the physician can serve each day. η is defined as the learning rate, which indicates the influence of the objective function reward observed last time on the next dual variable update. The learning-rate parameter η was selected through an empirical grid search on the validation set. We searched η over [0,1] with a step size of 0.1 and selected the value that achieved the highest average empirical CR. The selected η was then fixed and applied to the final test evaluation. It is treated as an empirical tuning parameter. Specifically, the algorithm initializes the defined dual variable $\alpha_{i,t}$ to 0. When the patient arrives at the platform, the physician-patient matching quality $r_{i,j}$ is first calculated based on the cosine similarity formula. Then, when the patient j meets the optimal function conditions, a physician will be assigned to the patient. Subsequently, the service capacity of physicians will change. When $r_{i,j}-\alpha_{i,t}\geq \text{0}$ is satisfied, the rule in step 7 is used to incrementally update the value of a dual variable, to continue to assign available physicians to subsequent arriving patients.

### Matching quality modeling

This study adopts the online rolling matching to assign appropriate physicians to arriving patients without making any assumptions about the future arrival. The recommendation decision mainly considers the capacity constraint, the patient arrival, and the matching quality. Previous studies on physician-patient matching have mostly focused on bilateral preferences and ignored cardinality utility. As online matching mechanisms play an increasingly important role in e-commerce, supply chain management, and healthcare management, it is necessary to better understand matching results from a utility perspective. It is very valuable to reasonably quantify the expected utility. To achieve an accurate calculation of matching quality, in-depth mining of patient questions and physician answers is required. Mining fine-grained semantic features is critical for natural language understanding.

Therefore, we use MPNet models from a fine-grained perspective to represent the matching quality. The cosine similarity is a standard function to quantify the difference between two vectors. The semantic similarity score can be calculated by the following:


$$\begin{array}{c} Simscore(x,y)=\frac{\sum_{i=\text{1}}^{n}(x_{i}\times y_{i})}{\sum_{i=\text{1}}^{n}(x_{i}{)}^{\text{2}}\times \sum_{i=\text{1}}^{n}(y_{i}{)}^{\text{2}}} \end{array}$$ (10)

Where x are the feature vectors of physician answers, and y are the feature vectors of patient questions.

## Results

### Data description

This study collected data from Haodf.com, the most popular online healthcare platform in China. The data collection and analysis procedures were conducted in accordance with the publicly available access conditions of Haodf.com. We constructed a dataset of 88,009 records spanning from January 1, 2019 to December 31, 2020. Only publicly available consultation information was used, and no private account information, contact information, or personally identifiable information was collected. Before constructing the experimental datasets, we removed records with missing key fields, filtered consultations by department, sorted patients by arrival time, and retained physicians satisfying the activity criterion. Because the platform contains physicians from many departments and online recommendation is typically specialty-specific, directly constructing a platform-wide matching problem over all registered physicians would not reflect the actual recommendation process and would also lead to unnecessary computational complexity. We chose the cardiovascular internal medicine (CIM) and hepatic surgery (HS) departments as our research objects, referring to them as the CIM dataset and HS dataset, respectively.

**Fig 2:**
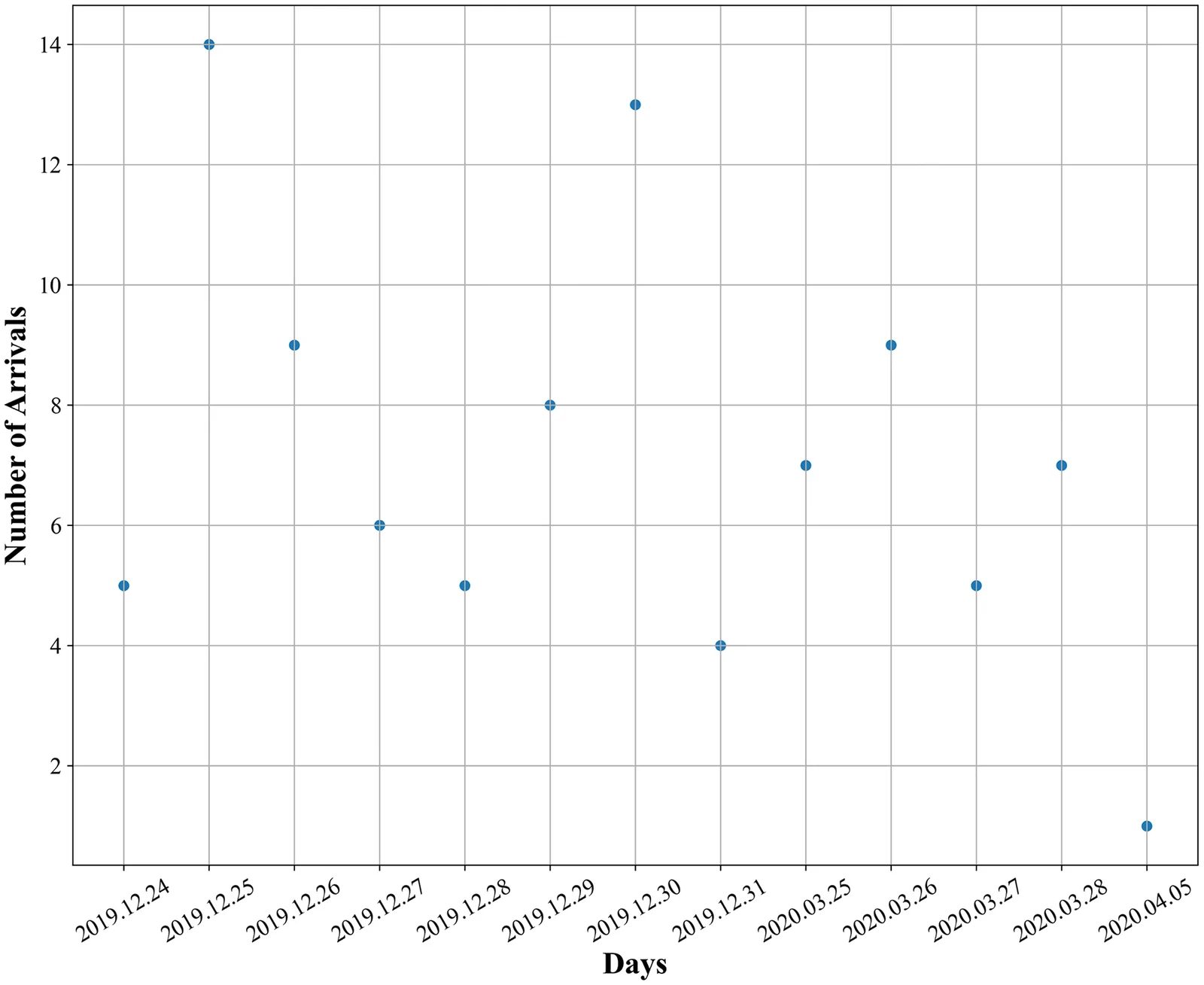
The distribution of patients on each day for randomly selected physicians.

In the above datasets, we can identify (i) the diagnoses or chief complaints of the patient, (ii) the arrival time of patients, and (iii) the previous answers posted by the physicians. For the physician selection in these two departments, we follow the principle of activity level. Physicians with low activity levels may have withdrawn from the platform. Thus, we chose physicians who answered questions more than 5 times over two years. Ultimately, our dataset included 60 highly active physicians and 1436 patients in the cardiovascular internal medicine department. Similarly, we have 36 physicians and 716 arriving patients in the hepatic surgery department. A basic description of datasets is provided in Table 1. Moreover, the dynamic arrival distribution of a physician in Fig 2 is displayed. This figure shows the number of patients a physician can treat in one day, and the high degree of uncertainty of patient arrival.

**Table 1:** Descriptive statistics of datasets.

Datasets	CIM	HS
Number of physicians	60	36
Number of patients	1436	716

Since the platform lacks specific information about the patient’s arrival, such as detailed time and minute data, days are chosen as the scheduling time. We estimate the capacity parameter by analyzing historical data. The estimated capacity should be interpreted as an observable proxy for platform-based service capacity rather than true latent physician capacity. Specifically, according to the arrival time of patients served by each physician, we can count the number of patients at each time point as the online service capacity. We constructed average, maximum, and minimum capacity settings based on historical daily consultation volumes. It is worth noting that the average value of capacity is rounded down. This is because our study addresses a physician dynamic recommendation problem under capacity constraints. If it is rounded up, it will be contrary to the research goal.

To valid the enhancement of semantic features, we constructed a dataset from January 2020 to December 2020. It includes 48,121 question-answering pairs after excluding the text whose response length was less than 15 characters. To label the answer quality, we organized six physicians to randomly evaluate the answer samples. To further verify the reliability of manual labeling, it is necessary to analyze the consistency between physicians. Fleiss’s Kappa coefficient is often adopted to test the consistency between multiple scorers. The calculated Fleiss’s Kappa value is 0.849. This shows that there is a high degree of consistency, which meets the requirements of repeatability and reliability of this experiment.

### Experiment setup

For the section on calculating the matching quality, the LDA model and MPNet model used in the experiment are the default parameters. The number of topics in the LDA model is specified as 10. It can ensure that the number of topics covers all patient questions while controlling the dimension of topic features, thus avoiding a reduction in computational efficiency. Here, we use the sentence-transformers repository to implement the MPNet model. The repository provides a variety of pre-training models to compute vector representations of text and images and achieves state-of-the-art performance in a variety of tasks. MPNet is a sentence-transformers model and it maps sentences to a 768-dimensional dense vector space. Detailed pre-training, fine-tuning, and hyperparameter settings for the MPNet model can be found at the following link: https://huggingface.co/sentence-transformers/all-mpnet-base-v2.

To verify the effectiveness of semantic enhancement, we selected the baseline models, namely K-Nearest Neighbor (KNN), Logistic Regression (LR), Support Vector Machine (SVM), Decision Tree (DT), Random Forest (RF), Gradient Boosting Decision Tree (GBDT), eXtreme Gradient Boosting (XGBoost), and Light Gradient Boosting Machine (LightGBM). All classification models are executed under default parameters, and the ratio of the training set and test set division is 8:2.

For the validation of the online rolling matching algorithm, we used the Gurobi solver under the 13.0.2 version. The physician-patient matching quality threshold is set at 0.75.

### Performance measure

This section adopts the evaluation indicators of classification tasks, including accuracy, precision, recall, F1, and Area Under the Curve (AUC) values. The total number of answers is represented as $num_{total}$, the amount of answer data correctly predicted by the model is defined as $num_{correct}$, the number of predicted high-quality answers is represented as $num_{predicted-high}$, the number of actual high-quality answers is defined as $num_{actual-high}$, and the number of high-quality answers correctly predicted by the model is defined as $num_{correct-high}$. The definitions of the above evaluation indicators are as follows:


$$\begin{array}{c} accuracy=\frac{num_{correct}}{num_{total}} \end{array}$$ (11)

$$\begin{array}{c} precision=\frac{num_{correct-high}}{num_{predicted-high}} \end{array}$$ (12)

$$\begin{array}{c} ecall=\frac{num_{correct-high}}{num_{actual-high}} \end{array}$$ (13)

$$\begin{array}{c} F\text{1}=\frac{\text{2}\times precision\times recall}{precision+recall} \end{array}$$ (14)

Competitive ratio (CR) is a commonly used metric to evaluate the performance of online algorithms. In this study, CR is used as an empirical online-to-offline performance ratio. Specifically, CR is defined as the objective value achieved by the online algorithm divided by the objective value of the offline optimal solution under the same observed arrival sequence and capacity setting. Therefore, CR is adopted to evaluate realized online performance relative to an offline benchmark, rather than to claim a worst-case theoretical guarantee under adversarial arrivals. The offline optimization problem is regarded as a benchmark to evaluate its performance. offline optimal objective value (OPT) represents the objective value of an optimal offline algorithm. And let online algorithm objective value (ALG) mean the expected value obtained by our method. We want to present an online algorithm, which is γ-competitive. A larger CR value means that the online algorithm can apply better to the maximization problem. The CR can be defined as follows:


$$\begin{array}{c} \gamma =inf\left\{\frac{ALG}{OPT}\right\} \end{array}$$ (15)

### Results of semantic representations

To examine the effect of semantic representations, we compared topic features, MPNet-based semantic features, and fused LDA-MPNet features using several common machine learning classifiers. As shown in Table 2, models incorporating MPNet-based semantic information generally achieve better classification performance than those using topic features alone. The fused LDA-MPNet features perform best across all five metrics for KNN, LR, and DT, and improve accuracy, precision, and AUC for SVM. For RF, only recall slightly decreases by 0.001, while the other metrics improve. GBDT, XGBoost, and LightGBM also show improvements in accuracy, recall, F1, and AUC after incorporating semantic features. These results suggest that MPNet-based contextual semantic representations provide substantial additional information beyond LDA topic features.

**Table 2:** Auxiliary evaluation of semantic representations in answer quality prediction.

Model	Feature	Accuracy	Precision	Recall	F1	AUC
KNN	LDA	0.792	0.969	0.803	0.878	0.765
	MPNet	0.798	0.980	0.799	0.881	0.848
	LDA_MPNet	**0.806**	**0.984**	**0.805**	**0.886**	**0.865**
LR	LDA	0.712	0.966	0.717	0.823	0.735
	MPNet	0.880	0.987	0.882	0.932	0.934
	LDA_MPNet	**0.889**	**0.989**	**0.891**	**0.937**	**0.937**
SVM	LDA	0.793	0.974	0.800	0.878	0.826
	MPNet	0.940	0.978	0.957	0.968	0.924
	LDA_MPNet	**0.939**	**0.980**	0.955	0.967	**0.932**
DT	LDA	0.897	0.964	0.924	0.944	0.714
	MPNet	0.880	0.966	0.904	0.934	0.726
	LDA_MPNet	**0.892**	**0.967**	**0.916**	**0.941**	**0.735**
RF	LDA	0.925	0.965	0.954	0.959	0.863
	MPNet	0.941	0.967	0.969	0.968	0.918
	LDA_MPNet	**0.941**	**0.969**	0.968	**0.968**	**0.924**
GBDT	LDA	0.873	0.981	0.881	0.928	0.884
	MPNet	0.882	0.985	0.887	0.933	0.922
	LDA_MPNet	**0.898**	0.983	**0.907**	**0.943**	**0.926**
XGBoost	LDA	0.906	0.971	0.927	0.948	0.875
	MPNet	0.939	0.973	0.961	0.967	0.919
	LDA_MPNet	**0.946**	0.972	**0.970**	**0.971**	**0.925**
LightGBM	LDA	0.903	0.974	0.921	0.947	0.887
	MPNet	0.922	0.979	0.937	0.958	0.925
	LDA_MPNet	**0.937**	0.976	**0.956**	**0.966**	**0.931**

The LDA-MPNet fusion results are used only as an auxiliary validation of semantic representation in the classification task. Experimental results (as shown in Table 3) also demonstrate that MPNet-only performance is the most competitive, further illustrating the crucial importance of contextual semantic representation for constructing matching quality. Using MPNet, the physician-patient matching quality matrix more accurately characterizes the contextual semantic relationship between patient needs and physician expertise. Compared to the coarse-grained topic representation of LDA, the matching quality ranking generated by MPNet is more stable and discriminative, making it easier for the online rolling matching to select a physician allocation scheme close to the offline optimum in dynamic arrival scenarios. Since MPNet-only achieves the best performance in the downstream matching task, the final online matching experiments use MPNet-based semantic representations to construct matching quality scores.

**Table 3:** Comparison of matching quality representations.

Datasets	Capacity	Models	Mean CR	Std.	95%CI
CIM	Average	LDA	0.868	0.0020	[0.8669, 0.8683]
		**MPNet**	**0.977**	0.0003	[0.9768, 0.9770]
		LDA-MPNet	0.929	0.0010	[0.9291, 0.9298]
	Maximum	LDA	0.924	0.0012	[0.9236, 0.9244]
		**MPNet**	**0.989**	0.0001	[0.9887, 0.9888]
		LDA-MPNet	0.962	0.0006	[0.9615, 0.9619]
	Minimum	LDA	0.855	0.0021	[0.8538, 0.8554]
		**MPNet**	**0.967**	0.0004	[0.9672, 0.9675]
		LDA-MPNet	0.917	0.0010	[0.9171, 0.9178]
HS	Average	LDA	0.862	0.0040	[0.8602, 0.8630]
		**MPNet**	**0.971**	0.0007	[0.9709, 0.9713]
		LDA-MPNet	0.927	0.0019	[0.9268, 0.9281]
	Maximum	LDA	0.947	0.001	[0.9466, 0.9473]
		**MPNet**	**0.990**	0.0003	[0.9897, 0.9899]
		LDA-MPNet	0.975	0.0006	[0.9752, 0.9756]
	Minimum	LDA	0.847	0.0035	[0.8457, 0.8482]
		**MPNet**	**0.966**	0.0006	[0.9661, 0.9665]
		LDA-MPNet	0.918	0.0020	[0.9172, 0.9186]

All metrics in the entire paper are rounded to three decimal places. To avoid small fluctuations being rounded to 0, the standard deviation and confidence interval are rounded to four decimal places.

### Results of dynamic physician recommendation

The experimental results of our method will be analyzed and discussed in detail. According to Table 4, in 30 repeated experiments, online rolling matching demonstrated high and stable CR across different capacity settings. Specifically, in the CIM dataset, the mean CRs for average, maximum, and minimum capacity settings were 0.977, 0.989, and 0.967, respectively; in the HS dataset, the corresponding mean CRs were 0.971, 0.990, and 0.966. Both datasets show that the CR was highest at the maximum capacity setting and relatively lower at the minimum capacity setting, indicating that the more sufficient the capacity, the easier it is for the online matching algorithm to obtain allocation results close to the offline optimum.

**Table 4:** Results of online rolling matching under different capacity.

Datasets	Capacity	Mean CR	Std.	95%CI
CIM	Average	0.977	0.0003	[0.9768, 0.9770]
	Maximum	0.989	0.0001	[0.9887, 0.9888]
	Minimum	0.967	0.0004	[0.9672, 0.9675]
HS	Average	0.971	0.0007	[0.9709, 0.9713]
	Maximum	0.990	0.0003	[0.9897, 0.9899]
	Minimum	0.966	0.0006	[0.9661, 0.9665]

Meanwhile, the standard deviations under each experimental setting are small, and the 95% confidence intervals are very narrow. This indicates that the proposed method has strong stability in different randomized repeated experiments and does not fluctuate significantly due to perturbations in patient arrival order.

### Comparison results

In comparative experiments, this paper compares the proposed method with five baselines: threshold policy, greedy policy, dynamic physician assortment, dynamic stochastic matching, and re-solving online matching. By comparing with these methods, the effectiveness of the proposed method can be more comprehensively evaluated.

(1) **Threshold policy** [8]: Given a constant ϕ⩾0, the threshold matches $n_{i}$ with the first arriving node n such that $\left(n_{i},n\right)⩽\phi$. If the condition is met, the threshold policy matches each arriving patient in turn with the physician list. Once the matching quality exceeds the set threshold, we assign the physician to the patient. Once a physician is assigned, they are removed from the list of available physicians. The policy will repeat this step until all patients are assigned.
(2) **Greedy policy** [7]: In each iteration, the greedy policy selects the maximum weight matching in the current state, to produce the optimal result. The choice made by it is the best in the current state. Moreover, the current decision will not affect the subsequent decision. Finally, it provides a locally optimal solution to the problem. In the context of physician-patient matching, the greedy policy is to allocate a suitable physician and specific service time to maximize their reward.
(3) **Dynamic physician assortment** [18]: Dynamic physician assortment is used to simulate patient selection behavior in online physician recommendation scenarios. For each arriving patient, the algorithm first constructs a Top-K candidate physician set from physicians with remaining capacity for the day, based on the patient-physician match quality. Subsequently, the algorithm converts the match quality in the candidate set into softmax selection probabilities, representing the probability of the patient’s preference for different physicians.
(4) **Dynamic stochastic matching** [57]: Dynamic stochastic matching is an online dynamic matching strategy that considers finite wait times and future demand pressure. Patients arrive dynamically and are added to the backlog, continuously participating in subsequent matching within the maximum wait period. The algorithm first estimates the patient arrival rate based on historical arrival data to characterize future demand pressure. For each currently matchable patient, the algorithm considers not only the initial matching quality but also calculates a capacity scarcity penalty based on the remaining capacity within the physician’s future window.
(5) **Re-solving online matching** [36]: The re-solving baseline is used to simulate a simple rolling re-optimization approach. Patients enter the system according to their actual arrival date; those not matched in time are added to the backlog and continue waiting on subsequent days. Each service day, the algorithm first collects the patients who have arrived but not yet been matched, and processes the backlog in a first-come, first-served (FIFO) order. For each patient awaiting matching, the algorithm selects the physician with the highest matching quality from the set of physicians with remaining capacity for that day. If the matching quality is below a preset minimum quality threshold, the patient remains in the backlog waiting for the next round of matching.

Based on the comparative experimental results in Table 5, the proposed method presented in this paper achieved the highest CR on both the CIM and HS datasets, with a narrow 95% confidence interval, indicating that the method not only has higher matching efficiency but also performs stably in repeated experiments. Based on simple baseline performance, threshold policy has the lowest CR at 0.923 on the CIM dataset and 0.894 on the HS dataset. This is because threshold policy only accepts a match when the matching quality meets the set threshold, potentially rejecting some still valuable but feasible matches. In contrast, greedy policy selects the physician with the highest match quality among currently available physicians at each decision, thus outperforming threshold policy on both datasets.

**Table 5:** Online rolling matching versus baseline models.

Datasets	Methods	CR	95%CI	t-statistic	Sig.
CIM	Threshold policy	0.923	[0.9224, 0.9229]	563.314	***
	Greedy policy	0.946	[0.9452, 0.9464]	135.661	***
	Dynamic physician assortment	0.969	[0.9687, 0.9690]	279.625	***
	Dynamic stochastic matching	0.964	[0.9635, 0.9637]	391.656	***
	Re-solving online matching	0.973	[0.9730, 0.9730]	588.454	***
	**Online rolling matching**	**0.988**	[0.9883, 0.9884]	**–**	**–**
HS	Threshold policy	0.894	[0.8925, 0.8951]	142.717	***
	Greedy policy	0.926	[0.9248, 0.9273]	101.879	***
	Dynamic physician assortment	0.947	[0.9469, 0.9477]	194.187	***
	Dynamic stochastic matching	0.957	[0.9572, 0.9577]	205.830	***
	Re-solving online matching	0.972	[0.9723, 0.9723]	260.238	***
	**Online rolling matching**	**0.988**	[0.9876, 0.9878]	**–**	**–**

Note: ***: p < 0.001, **: p < 0.01, *: p < 0.05.

Further comparison with stronger baselines reveals that dynamic physician assortment, dynamic stochastic matching, and re-solving online matching generally outperform threshold and greedy. Specifically, dynamic physician assortment achieves CRs of 0.969 and 0.947 on CIM and HS, respectively, demonstrating that modeling with a Top-K physician set and patient selection probability can improve recommendation quality. Dynamic stochastic matching achieves CRs of 0.964 and 0.957 on the two datasets, respectively, showing that considering future demand pressure and physician scarcity helps improve dynamic matching results. Re-solving online matching performs closest to the proposed method, achieving CRs of 0.973 and 0.972 on CIM and HS, respectively, indicating that backlog-based periodic re-optimization can effectively utilize the current system state and remaining physician capacity.

Although these stronger baselines are already highly competitive, our method still achieves superior performance. Significance tests further support these conclusions. The proposed method’s improvement relative to each baseline reaches a significant level with a p-value less than 0.001, indicating that these performance differences are not caused by random fluctuations but are statistically significant in repeated experiments. Overall, the experimental results validate the effectiveness and stability of the proposed method on different departmental datasets.

## Discussion

### Sensitivity analysis

**Fig 3:**
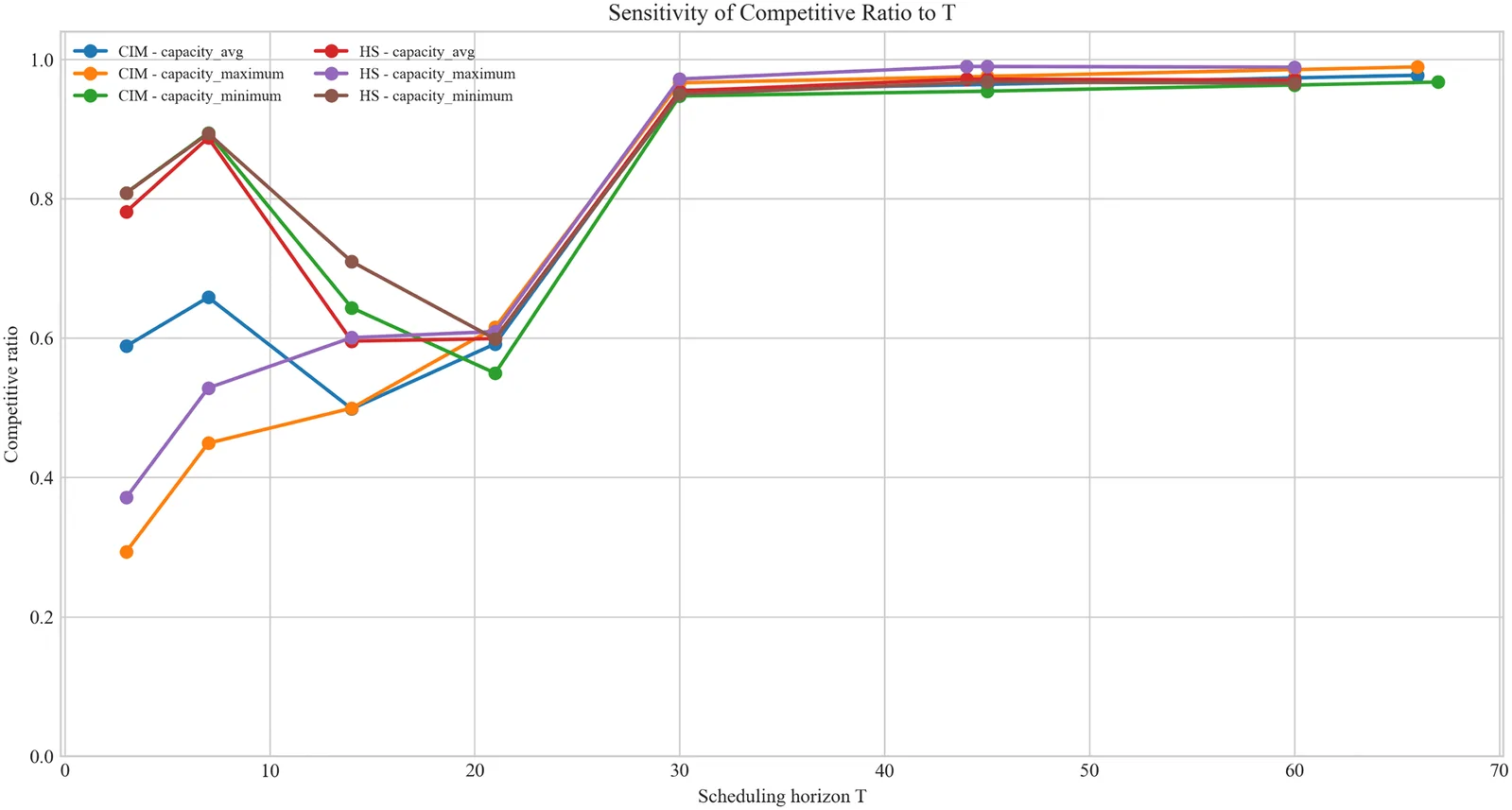
Sensitivity analysis of scheduling period.

This section conducts a sensitivity analysis on the impact of the scheduling period and service capacity on the CR. Fig 3 shows the CR trend of online rolling matching under different scheduling periods T, and further compares the average, maximum, and minimum capacity settings. In the CIM dataset, when T is small, the CR fluctuates significantly under different capacity settings, especially under the average and maximum capacity settings. The shorter scheduling period limits the algorithm’s ability to coordinate physician resources. This indicates that when the rolling window is long enough, the algorithm can make fuller use of future service capacity, thus obtaining matching results closer to the offline optimum. A similar trend can be observed in the HS dataset. Compared to the short-period setting, the longer scheduling period provides more room for patient delay decisions and physician capacity coordination, thereby improving the overall matching quality.

Overall, sensitivity analysis shows that the scheduling period T is a crucial parameter affecting the performance of online rolling matching. An excessively short scheduling period limits the algorithm’s utilization of future capacity, resulting in a lower CR. However, once T reaches a certain level, the algorithm’s performance tends to stabilize, and further extending the scheduling period yields limited marginal benefits. Therefore, a moderate rolling scheduling window can achieve a good balance between matching performance and computational cost.

We will examine the impact of the capacity change in further detail. According to the sensitivity analysis of service capacity results in Table 6, after increasing capacity scale by 10%, 20%, and 50%, respectively, online rolling matching maintains a high and stable CR on both the CIM and HS datasets. This indicates that when the actual physician service capacity is higher than the historical estimated capacity, the proposed method can still obtain matching results close to the offline optimum.

**Table 6:** Results of online rolling matching in different capacity scale.

Datasets	Capacity scale	Mean CR	Std.	95%CI
CIM	Scale = 1.1	0.992	0.0001	[0.9915, 0.9916]
	Scale = 1.2	0.992	0.0001	[0.9916, 0.9917]
	Scale = 1.5	0.992	0.0000	[0.9923, 0.9924]
HS	Scale = 1.1	0.992	0.0002	[0.9920, 0.9921]
	Scale = 1.2	0.992	0.0002	[0.9921, 0.9922]
	Scale = 1.5	0.993	0.0002	[0.9927, 0.9929]

Meanwhile, the proposed method exhibits very narrow 95% confidence intervals under different capacities, indicating strong stability in repeated runs. As the capacity increases from 1.1 to 1.5, the CR slightly improves, suggesting that a more sufficient capacity makes it easier to alleviate matching conflicts caused by capacity constraints, thereby further improving overall matching efficiency. This result demonstrates that even if we underestimate the actual capacity of physicians, the proposed method still exhibits good robustness under different capacity expansion scenarios.

### Robustness analysis

To examine the robustness of the proposed method under physician-side capacity uncertainty, we perturb the estimated physician capacity by introducing multiplicative random noise. Specifically, the perturbed capacity of physician j in period t is defined as:


$$\begin{array}{c} {\tilde{c}}_{i,t}=c_{i,t}(\text{1}+\epsilon ),\epsilon \sim uniform(-\rho ,\rho ) \end{array}$$ (16)

where $c_{i,t}$ denotes the estimated service capacity, ${\tilde{c}}_{i,t}$ denotes the perturbed capacity, and ρ controls the noise intensity.

We set ρ=0.1,0.2,0.3 to represent mild, moderate, and relatively strong capacity-estimation noise, corresponding to ±10%, ± 20%, and ±30% perturbations of service capacity. This design allows us to evaluate whether the proposed method remains effective when physician capacity information is incomplete or noisy.

Table 7 shows that, on both the CIM and HS datasets, the proposed method maintains the highest CR across all capacity perturbation levels as the perturbation ratio increases from 0.1 to 0.3. In terms of method performance, the threshold policy and greedy policy remain relatively weak baselines under capacity perturbation, suggesting that simple rule-based strategies are more susceptible to matching-opportunity losses in capacity-uncertain environments. In contrast, the other three baseline models show more stable performance, with re-solving online matching being the closest baseline to the proposed method on both datasets. This indicates that periodic re-optimization and the backlog mechanism can effectively mitigate the impact of capacity perturbation.

**Table 7:** Robustness results of capacity noise.

Datasets	ρ	Methods	CR	95%CI	t-statistic	Sig.
CIM	ρ = 0.1	Threshold policy	0.923	[0.9224, 0.9230]	469.825	***
		Greedy policy	0.946	[0.9452, 0.9465]	136.329	***
		Dynamic physician assortment	0.969	[0.9687, 0.9690]	264.521	***
		Dynamic stochastic matching	0.964	[0.9634, 0.9638]	260.926	***
		Re-solving online matching	0.973	[0.9730, 0.9731]	535.807	***
		**Online rolling matching**	**0.988**	[0.9883, 0.9884]	–	**–**
	ρ = 0.2	Threshold policy	0.923	[0.9223, 0.9234]	248.429	***
		Greedy policy	0.946	[0.9450, 0.9465]	121.163	***
		Dynamic physician assortment	0.969	[0.9685, 0.9688]	258.821	***
		Dynamic stochastic matching	0.963	[0.9631, 0.9638]	142.910	***
		Re-solving online matching	0.973	[0.9729, 0.9730]	532.830	***
		**Online rolling matching**	**0.988**	[0.9882, 0.9884]	–	–
	ρ = 0.3	Threshold policy	0.922	[0.9214, 0.9229]	187.405	***
		Greedy policy	0.945	[0.9447, 0.9462]	116.913	***
		Dynamic physician assortment	0.968	[0.9682, 0.9688]	218.175	***
		Dynamic stochastic matching	0.963	[0.9627, 0.9637]	106.781	***
		Re-solving online matching	0.973	[0.9726, 0.9729]	430.244	***
		**Online rolling matching**	**0.988**	[0.9879, 0.9883]	–	–
HS	ρ = 0.1	Threshold policy	0.894	[0.8925, 0.8950]	146.593	***
		Greedy policy	0.926	[0.9246, 0.9271]	101.780	***
		Dynamic physician assortment	0.947	[0.9469, 0.9477]	193.543	***
		Dynamic stochastic matching	0.958	[0.9573, 0.9578]	199.477	***
		Re-solving online matching	0.972	[0.9723, 0.9723]	265.863	***
		**Online rolling matching**	**0.988**	[0.9876, 0.9878]	–	–
	ρ = 0.2	Threshold policy	0.894	[0.8926, 0.8953]	144.027	***
		Greedy policy	0.926	[0.9243, 0.927]	95.182	***
		Dynamic physician assortment	0.947	[0.9467, 0.9476]	186.045	***
		Dynamic stochastic matching	0.957	[0.9569, 0.9581]	114.047	***
		Re-solving online matching	0.972	[0.9722, 0.9724]	244.759	***
		**Online rolling matching**	**0.988**	[0.9875, 0.9878]	–	–
	ρ = 0.3	Threshold policy	0.892	[0.8909, 0.8937]	133.247	***
		Greedy policy	0.924	[0.9226, 0.9261]	78.076	***
		Dynamic physician assortment	0.947	[0.9462, 0.9473]	169.169	***
		Dynamic stochastic matching	0.958	[0.9566, 0.9590]	62.212	***
		Re-solving online matching	0.973	[0.9721, 0.9730]	191.503	***
		**Online rolling matching**	**0.988**	[0.9873, 0.9881]	**–**	**–**

Note: ***: p < 0.001, **: p < 0.01, *: p < 0.05.

As the perturbation ratio increases, the CR values of most baselines show slight decreases or fluctuations, whereas the decrease in online rolling matching is minimal. Moreover, its advantage over all baselines is significant at the 0.001 level. This indicates that the proposed method can effectively balance current matching benefits with future capacity preservation when capacity information is noisy, thereby maintaining online matching performance close to the offline optimum.

Table 8 compares the robustness of each method under temporary physician unavailability scenarios. The experiment included three physician unavailability percentages: 5%, 10%, and 20%, to simulate supply-side disturbances in real-world platforms, such as physicians temporarily logging off, taking leave, or scheduling conflicts. The results show that as the unavailability percentage increases, the CR of each method decreases to varying degrees, indicating that reduced physician availability weakens the overall performance of the online matching system.

**Table 8:** Robustness results of physician unavailable rates.

Datasets	Unavailable	Methods	CR	95%CI	t-statistic	Sig.
CIM	Rate = 0.05	Threshold policy	0.921	[0.9200, 0.9212]	241.698	***
		Greedy policy	0.943	[0.9427, 0.9437]	171.460	***
		Dynamic physician assortment	0.967	[0.9671, 0.9674]	237.545	***
		Dynamic stochastic matching	0.944	[0.9433, 0.9438]	369.634	***
		Re-solving online matching	0.972	[0.9719, 0.9721]	336.407	***
		**Online rolling matching**	**0.987**	[0.9872, 0.9874]	–	**–**
	Rate = 0.1	Threshold policy	0.918	[0.9176, 0.9191]	214.944	***
		Greedy policy	0.941	[0.9398, 0.9414]	122.434	***
		Dynamic physician assortment	0.966	[0.9654, 0.9659]	210.859	***
		Dynamic stochastic matching	0.942	[0.9418, 0.9425]	299.333	***
		Re-solving online matching	0.971	[0.9709, 0.9712]	374.201	***
		**Online rolling matching**	**0.986**	[0.9863, 0.9866]	–	–
	Rate = 0.2	Threshold policy	0.914	[0.9133, 0.9151]	145.281	***
		Greedy policy	0.934	[0.9333, 0.9346]	143.451	***
		Dynamic physician assortment	0.962	[0.9613, 0.9618]	234.919	***
		Dynamic stochastic matching	0.939	[0.9387, 0.9397]	202.299	***
		Re-solving online matching	0.969	[0.9683, 0.9688]	232.083	
		**Online rolling matching**	**0.984**	[0.9836, 0.9841]	–	–
HS	Rate = 0.05	Threshold policy	0.890	[0.8885, 0.8912]	148.337	***
		Greedy policy	0.920	[0.9187, 0.9213]	101.780	***
		Dynamic physician assortment	0.944	[0.9435, 0.9443]	189.005	***
		Dynamic stochastic matching	0.933	[0.9321, 0.9331]	198.479	***
		Re-solving online matching	0.971	[0.9707, 0.9710]	212.936	***
		**Online rolling matching**	**0.986**	[0.9858, 0.9862]	–	–
	Rate = 0.1	Threshold policy	0.885	[0.883, 0.887]	110.966	***
		Greedy policy	0.913	[0.9108, 0.9148]	78.005	***
		Dynamic physician assortment	0.940	[0.9400, 0.9410]	165.921	***
		Dynamic stochastic matching	0.930	[0.9291, 0.9304]	184.547	***
		Re-solving online matching	0.969	[0.9690, 0.9695]	182.851	***
		**Online rolling matching**	**0.984**	[0.9838, 0.9844]	–	–
	Rate = 0.2	Threshold policy	0.873	[0.8706, 0.8756]	95.572	***
		Greedy policy	0.900	[0.8952, 0.9003]	71.735	***
		Dynamic physician assortment	0.935	[0.9341, 0.9352]	160.134	***
		Dynamic stochastic matching	0.927	[0.9264, 0.9280]	128.946	***
		Re-solving online matching	0.967	[0.9666, 0.9673]	125.671	***
		**Online rolling matching**	**0.981**	[0.9807, 0.9815]	**–**	**–**

Note: ***: p < 0.001, **: p < 0.01, *: p < 0.05.

Nevertheless, our method maintained the highest CR across all unavailability percentages on both datasets. In the CIM dataset, the CR of the proposed method decreased from 0.987 to 0.984 as the unavailability percentage increased from 5% to 20%; in the HS dataset, the CR decreased from 0.986 to 0.981, indicating that the method remains robust even under scenarios where physicians are temporarily unavailable. Based on baseline performance, threshold policy and greedy policy are more sensitive to physician unavailability, especially in the HS dataset. In contrast, the other three methods are more stable. In summary, these results suggest that online rolling matching can better adapt to temporary physician unavailability and maintain high matching performance.

### Theoretical implications

This study provides a dynamic physician recommendation method based on the primal-dual framework for online healthcare platforms. First, we allocate physicians from the perspective of resource constraints. Most of the existing physician recommendation research focuses on patient preferences, privacy, trust, etc., ignoring the characteristics of physician resources. Here, we use LP to build a physician-patient matching model. Secondly, we adopt advanced pre-trained models to calculate the matching quality of physician-patient. Previous studies have ignored the importance of deep semantic features. In short, our study supplements dynamic recommendations to a certain extent. This study also provides a new perspective on dynamic recommendation.

### Practical implications

Dynamic physician recommendation enhances the accuracy of physician-patient matching. The recommendation system can help patients find the physicians who best meet their needs, which not only improves the treatment effect but also promotes the establishment of trust between physicians and patients. However, the medical scenario differs from general online matching platforms. Patient waiting involves not only efficiency trade-offs but also safety, fairness of waiting, and informed choice. Therefore, the design of the delayed decision-making mechanism must not only focus on matching efficiency but also clarify its scope of application and ethical constraints.

Specifically, this mechanism should primarily be applied to non-urgent, low-risk online consultation scenarios, such as routine consultations and follow-up visits. It should be noted that due to limitations in data availability, this paper only obtains daily-level patient arrival and physician consultation information. Therefore, “day” is used as the time unit for rolling matching in the experiment. However, in real-world platform deployments, delayed decision-making should not be interpreted as necessarily postponing patients to the next day, but should be implemented based on more granular real-time data, such as dynamically updating patient queues, physician availability, and matching results on a minute or hourly basis.

In practical deployment, the platform should set a maximum waiting time, transparently informing patients of the expected waiting time, and retaining their right to choose immediate matching. Simultaneously, a risk triage mechanism should be introduced, prioritizing matching patients with urgent symptoms, high-risk profiles, or special vulnerabilities, or triggering manual review. These safeguards can improve matching efficiency while reducing the potential impact of delayed decisions on patient safety, fairness, and their right to choose.

## Conclusion

In this paper, we transform the problem of dynamic physician recommendation into one of online physician-patient matching. When a patient arrives at the platform, the corresponding practitioner should immediately make decisions regarding physician allocation and service time. Considering the uncertainty of patients and the dynamic capacity constraints of physicians, we construct an LP model based on a primal-dual framework. Then, we present an online rolling matching algorithm for physician-patient matching. Regarding the calculation of matching quality, we adopt MPNet model. Finally, the effectiveness of the proposed method is validated using large-scale real-world datasets collected from one of the largest online healthcare platforms in China. The experimental results show that the proposed method achieves superior performance compared with the baseline methods.

There are still some limitations in our research, which also suggest future research directions. First, the current framework relies on physicians’ historical consultations to construct expertise representations, which may lead to a cold-start problem for new physicians. Future research could use profile-level information to initialize physician representations and update them as more consultation records accumulate. Second, physician capacity in this study is estimated from historical consultation volume and should be interpreted as an observable proxy for platform-based service capacity rather than true capacity. Such volume may reflect not only supply-side capacity but also demand-side exposure, such as patient demand, platform ranking, visibility, and historical popularity. Future research could incorporate more direct supply-side information, including offline schedules, real-time online status, and response-time data, to estimate physician capacity more accurately. Finally, the evaluation based on two departments from a single platform has limited external validity. Future research should validate the proposed framework using multi-specialty and multi-platform datasets.
